# Promotive and preventive interventions for adolescent mental health in Sub-Saharan Africa: a combined scoping and systematic review

**DOI:** 10.1136/bmjph-2023-000037

**Published:** 2023-12-05

**Authors:** Maaike Seekles, Fantacy Twagira, Ali Alam, Angela Obasi

**Affiliations:** 1 Department of International Public Health, Liverpool School of Tropical Medicine, Liverpool, UK; 2 Department of Haemato-Oncology, Barts Health NHS Trust, London, UK

**Keywords:** Public Health, Preventive Psychiatry, Community Health

## Abstract

**Introduction:**

Poor mental health in adolescence is associated with mental, physical and social problems in later life. Adolescence is, therefore, a critical time for promoting mental well-being and preventing mental illness, particularly in sub-Saharan Africa, where adolescents are exposed to a multitude of risk factors for poor mental health. This review aimed to map the current use, effectiveness and cultural sensitivity of promotive/preventive adolescent mental health interventions in the region.

**Methods:**

A combined scoping and systematic review was conducted using the Arksey and O’Malley framework through searches in MEDLINE, CINAHL, Global Health, PsycINFO and the Cochrane Database of Systematic Reviews, covering January 2000 to December 2021.

**Results:**

This review identified 79 papers, related to 61 unique interventions. Only five universal, school-based programmes were identified; most studies targeted orphans or HIV positive adolescents. Psychosocial interventions—aimed at strengthening knowledge, expression and psychosocial skills—produced mixed results. Structural interventions were often community-based and had limited psychosocial programming. Those that focused on HIV prevention, gender equity and parenting also produced mixed results; evidence was strongest for economic-livelihood programmes. Few studies described cultural sensitivity in detail. Some explained how the intervention aligned with local worldviews/values; had context-specific content; were based on explorations of relevant concepts; or integrated spiritual/cultural practices.

**Conclusion:**

Preventive/promotive interventions for adolescent mental health in sub-Saharan Africa are limited in terms of geographical spread, but broad in terms of intervention types. Targeted approaches reflect realities that adolescents in the region face in relation to socioeconomic deprivation, family disruption and poor physical health. Yet, universal interventions that focus on general well-being are limited and lack a consideration of contemporary developments in the region such as increased social media use, suicide and obesity. Economic livelihood interventions showed most consistent evidence of effectiveness. Future studies could do more to consider/report cultural sensitivity.

**WHAT IS ALREADY KNOWN ON THIS TOPIC**

- A recent scoping review provided a high-level overview of study characteristics and intervention settings, populations, outcomes and providers of all types of adolescent mental health interventions in sub-Saharan Africa.

**WHAT THIS STUDY ADDS**

- This review adds a more in-depth exploration of different types of promotive/preventive interventions that were used; a systematic, at-a-glance overview of outcomes and effectiveness of interventions; and examples of how researchers tried to make interventions responsive to local/cultural context.

**HOW THIS STUDY MIGHT AFFECT RESEARCH, PRACTICE OR POLICY**

- This review informs further research and practice by highlighting impactful interventions, in addition to identifying gaps related to longitudinal study designs and universal interventions that include approaches related to suicide prevention, lifestyle (exercise/nutrition) and social media.

## Background

 Adolescent mental health (AMH) is increasingly recognised as a priority for global health and development. Mental health conditions are among the leading causes of disability in young people aged between 10 and 24 years^1^ and an estimated 63% of all mental disorders start before the age of 25.^2^ Poor AMH is associated with a range of health and social problems, such as physical ill health, underachievement in education and employment, poor interpersonal relationships and increased risk taking.^3 4^ The effects of these problems often persist throughout the life course and have serious implications.^5^ Consequently, adolescence—defined as a phase of life from ages 10 to 19^4^—is a critical time to implement interventions aimed at promoting mental well-being and preventing mental illness. This is particularly important in sub-Saharan Africa (SSA), where those under 25 make up around 65% of the population.^6^ Adolescents in this region may be particularly at risk of mental disorders when they are exposed to risks factors for adverse AMH such as poverty or marginalisation.^4^ Although limited, available evidence suggests that AMH conditions are highly prevalent in SSA, with median point prevalence rates of 27% for depression and 30% for anxiety disorders.^7 8^

Since AMH is affected by a wide range of biopsychosocial factors,^4^ promotive/preventive interventions can focus on a large variety of topics. The concepts of mental health promotion and prevention are intrinsically linked, and interventions often have overlapping goals and core components. Interventions can be categorised into biological (eg, pharmacotherapy); psychosocial (interpersonal or informational activities that use psychological, behavioural and/or social approaches to equip adolescents with psychosocial skills) or structural interventions (addressing environmental, social and/or economic risk factors of poor AMH).^4^ Systematic reviews highlight (a combination of) components used in psychosocial promotive/preventative interventions, including elements based on cognitive–behavioural therapy (CBT); social and emotional learning; positive psychology; mindfulness and mental health literacy.^9 10^ Structural interventions include programmes that focus on parenting practices, housing, poverty alleviation and/or access to health services,^11 12^ which do not necessarily include mental health programming content. Evidence of effectiveness of interventions^1013^,^15^ has informed recently published ‘WHO guidelines on mental health promotive and preventive interventions for adolescents’.^4^ However, most of this evidence stems from high-income countries (HICs) with individualistic societies^16^,^18^; few interventions have been implemented in lower-income and middle-income countries (LMICs).^19 20^ Intervention transferability from HIC to LMICs is context dependent. People in SSA often have cultural explanatory models for mental disorders that generally differ from those living in HICs and can include a belief in activities of spiritual and supernatural powers as cause for poor mental health.^21^ Local understandings of what constitutes mental health and social factors such as stigma, negative attitudes towards mental ill health, and collectivistic societal values,^22^ may impact the appropriateness and effectiveness of interventions developed in HICs in this setting. Therefore, it is important that interventions used in SSA are developed or adapted through a process of culturally informed research that explores relevant local concepts, to ensure context responsiveness and sensitivity.^23^

The use, cultural sensitivity and effectiveness of AMH promotion and prevention interventions in SSA is currently unclear. In response, the current review addressed the following, purposively broad, research question: ‘What is known from the existing literature about promotive or preventive interventions for AMH in SSA?’ Since the submission of our protocol,^24^ a scoping review examining all types of AMH interventions (including treatment interventions) in SSA has been published, which provided a high-level overview of study characteristics, intervention settings, populations, outcomes and providers.^19^ The current review adds to this a more in-depth description of promotion/prevention intervention types, an exploration of cultural sensitivity and a systematic review of effectiveness (including risk-of-bias assessments).

## Methodology

### Overview

This review was conducted in accordance with the Arksey and O’Malley framework,^25^ complemented by the Joanna Briggs Institute methodology^26^ for scoping reviews. The review process has been reported following the Preferred Reporting Items for Systematic Reviews and Meta-Analyses (PRISMA) Extension for Scoping Reviews guidelines.^27^ The methodology was specified in advance and published as a protocol.^24^ The systematic review has been registered on Prospero, ID: CRD42021297293.

### Patient and public involvement

Patients were not involved in the review process and drafting of this paper.

### Search strategy

Systematic searches were undertaken in the following databases: MEDLINE, CINAHL, Global Health, PsycINFO and the Cochrane Database of Systematic Reviews. Key search terms, including synonyms and medical subject headings were entered. Online supplemental file 1 contains the search terms for all databases. Databases were searched for entries from January 2000 to the 31 December 2021, without language restrictions, since MS and FT had joint proficiency in French, Italian, Spanish, Dutch and German.

### Inclusion criteria

The scoping review component included evaluation studies and formative studies and/or protocols linked to included evaluation studies; the systematic review component included randomised-controlled trials (RCTs) only. Table[Table T1] 1 provides a summary of the applied inclusion criteria and relevant definitions; further details and justification for these criteria can be found in the protocol.^24^

**Table 1 T1:** Inclusion criteria

	Included	Definition	Excluded
Study population	Adolescents	Individuals aged between 10 and 19 years^4^	Studies with less than 75% adolescents Studies with mean/median age<10 or >19
Intervention type	Promotion Universal prevention Selective prevention Psychosocial/structural intervention	Non-pharmacological interventions aimed at the general population that has not been identified based on risk (universal), or interventions targeted at subpopulations identified as being at elevated risk for a disorder (selective), but who are not selected for study inclusion based on symptoms or diagnosis of disorder.^4^	Indicated prevention Treatment intervention Substance (mis)use intervention (as recently reviewed in references 110 120) Pharmacological intervention.
Context	Studies in any sub-Saharan Africa (SSA) country (or at least one SSA country for multicountry studies).	Countries of SSA as defined by The World Bank, using both French and English country names.	Studies in (post-)conflict or humanitarian settings (as recently reviewed in references 121).
Outcome (evaluation studies only)	Adolescent mental health outcomes	For promotion interventions, these include indicators of positive mental health and emotional, psychological or social well-being, such as self-esteem, self-efficacy, coping skills, resilience, emotional well-being. For prevention interventions, target outcomes are indicators of negative mental health such as psychological distress and mental health disorders as listed in the Diagnostic and Statistical Manual of Mental Disorder, such as depression, anxiety and suicidal behaviour.	Mental health outcome not reported Outcome for adolescents not reported.
Publication date	After 1 January 2000	–	Before 1 January 2000
Language	Any language	–	–
Study design	Any evaluation study design Formative, intervention development studies, linked to published evaluation study Protocol papers of evaluation studies, if evaluation study not yet published.	–	Literature reviews excluded after citation search Interventions of which no evaluation exist.

### Screening and selection

**Figure 1:**
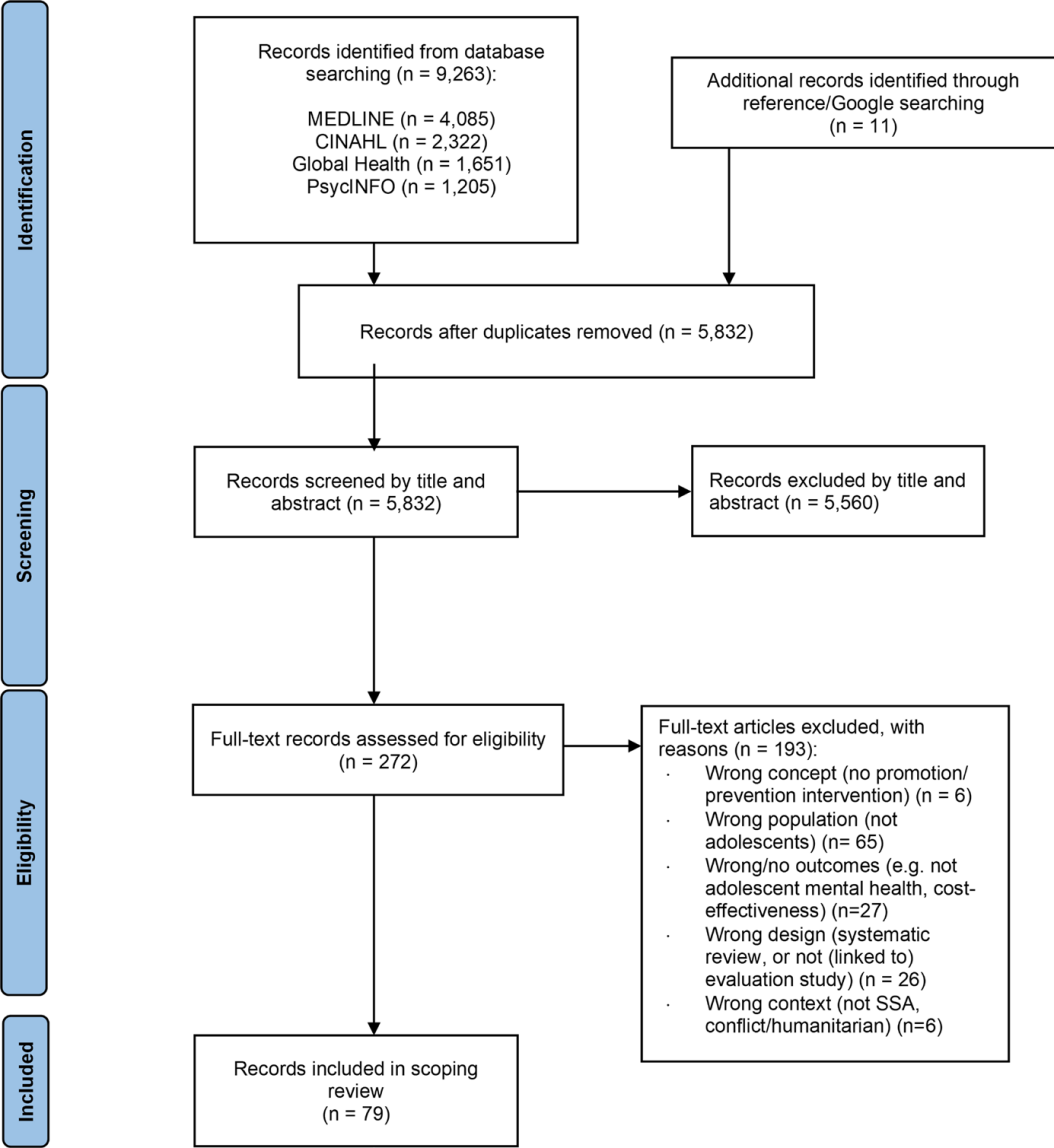
PRISMA flow diagram for inclusion of articles

Identified citations were imported into EndNote V.X9^28^ and duplicates removed; 5832 unique citations were imported into Rayyan Software.^29^ Title and abstract screening was done independently by two reviewers (MS and FT), following a pilot test whereby 50 articles were screened and inconsistencies between reviewer decisions were discussed. Full-text articles were reviewed by the same reviewers, apart from three papers written in French, which were read by FT only. Full texts of three potentially relevant articles were obtained via interlibrary loans. Disagreements about inclusion were resolved through discussions with a third reviewer (AO). The evaluation resulted in the inclusion of 79 eligible articles (figure 1).

### Data extraction and analysis

Data were extracted in duplicate and added to a data charting table in Microsoft Excel. MS performed a check for discrepancies between extracted data. This was an iterative process and categories were added as familiarity with the literature increased. Data on study characteristics were analysed descriptively and a numerical summary was provided. Studies were further categorised according to intervention type (psychosocial vs structural), and core focus/component (eg, bereavement, literacy, HIV prevention). Included studies were highly heterogeneous; in response, a narrative summary was provided under each category to give the reader an understanding of different intervention populations, types and outcomes.

The systematic review of intervention effectiveness only included results from RCTs. A tabular overview was created to accompany a narrative synthesis. Where possible, effect sizes (Cohen’s d) were calculated. Bias was assessed using the Cochrane risk of bias tool.^30^ A meta-analysis was not possible due to the heterogeneity of interventions.

Specific mentions of cultural tailoring or cultural sensitivity of interventions were summarised guided by the definition of Resnicow *et al*^31^ to differentiate between ‘surface-structure’ and ‘deep-structure’ adaptations. Surface-structure adaptations are those that make intervention materials and messages fit better with observable, ‘superficial’ characteristics of the target population. Deep-structure adaptations address core cultural values or ethnic, historical, social or environmental factors that may influence specific behaviours.

## Results

This review included 79 records, related to 61 interventions (table 2). To not distort findings, the count of primary studies was used in this analysis, unless stated otherwise.

**Table 2:** Included psychosocial interventions and core components

			Psychosocial components				Structural components			
**Intervention name**	**Evaluation type and country**	**Target group and setting (if universal**)	**Creative expressive**	**MHliteracy**	**DidacticSEL/CBT**	**Bereavement/ trauma**	**HIV prevention/SRHR**	**Family strength/ caregiver**	**Economic livelihood**	**IPV/gender equality**
Psychosocial interventions										
MH literacy										
n/a: MH literacy^32^	Waitlist control,Nigeria	UniversalSchool based		C						
n/a: MH training^33^	Non-randomised control, Nigeria	Universalschool based		C						
Awareness campaign^34^	Pre–post, Uganda	Universalcomm based		C						
Creative expressive										
Memory book^37^	Qualitative, SA	OVC	C			x				
Memory work therapy^38^	RCT,Tanzania	OVC	C			x				
n/a: Emotional writing^39^	RCT,Rwanda	OVC	C			x				
Playing to Live^35^	Pre–post, Liberia	Ebola affected	C		x	x				
Comfort 4 Kids^36^	Pre–post, Liberia	Ebola affected	C		x	x				
Psychosocial skills										
Shamiri digital^40^	RCT,Kenya	Universaldigital	x		C					
Shamiri^41^	Protocol, Kenya	Universalschool based	x		C					
ERASE stress prosocial^45^	RCT,Tanzania	Universalschool based			C					
n/a: resiliency programme^43^	RCT,SA	Universalschool based			C					
Youth first^46^	Pre–post,Kenya	Universalschool based			C					x
Living well programme^42^	Waitlist control,Uganda	Universalschool based			C					
Resourceful Adolesc Programme^44^	RCT,Mauritius	Universalschool based			C					
Child resilience programme^47^	Pre–post,Ethiopia	OVC		x	C					
Balekane EARTH^53^	Pre–post,Botswana	OVC	x		C	x		x		
Peer-support intervention^52^	RCT,Uganda	OVC	x		x		x			
Life-skills and psychoeducation^50^	RCT,Kenya	OVC		x	C					
n/a: resilience training^48 49^	Pre–post,Nigeria	OVC	x		C					
Ark for children^54^	Qualitative,Botswana	OVC			x	C				
Read me to resilience^51^	Qualitative,SA	OVC			C					
Thinking group^55^	Waitlist control, Nigeria	Aggressive males			C					
PAM programme^56 107^	RCT,SA	Visually impaired	x		C					
Psychosocial skills+bereavement										
Better accept reality^57 122^	Qualitative,SA	OVC	x		x	C				
Abangane^60^	RCT, SA	Female OVC	x	x	x	C				
n/a: bereavement intervention^59^	Qualitative,Zimbabwe	ALHIV	x			C				
n/a: interpersonal group therapy^58^	RCT,SA	OVC			x	C				
Psychosocial skills+HIVprevention/health promotion										
MAD about Arts^61^	Non-randomised control, SA	Universalcommunity based	x		C		x			
n/a: Counselling^62^	Pre–post,Ethiopia	Migrants	x		C		x			
n/a: Whatsapp group^63^	Pre–post,Kenya	ALHIV			C		x			
Sauti ya Vijana^64 65^	RCT,Tanzania	ALHIV		x	C	x	x	x		
Vhutshilo^67^	Longitudinal post-test, SA	Universalschool based			X	C	x			
Young citizens programme^66^	RCT,Tanzania	Universalcommunity based		C			x			

ALHIVadolescents living with HIVCcore intervention componentCBTcognitive–behavioural therapyIPVintimate partner violenceMHmental healthn/ano intervention nameOVCorphans and vulnerable childrenRCTrandomised controlled trialSASouth AfricaSELsocial emotional learningSRHRsexual and reproductive health and rightsxintervention component

Sixty-nine papers were intervention evaluations, including 39 (57%) RCTs. There were seven formative papers and two RCT protocols. The search identified no papers published before 2007; 75% of papers were published since 2015. Interventions were implemented in 14 out of 46 countries, with the majority implemented in South Africa (n=17, 28%), Kenya (n=10, 16%) or Uganda (n=10, 16%). The same intervention was never implemented in more than one country.

Seventeen interventions were universal, of which 10 were school-based. The remaining 44 interventions targeted a specific population; mainly orphans/vulnerable children (OVCs, n=23, 38%) and adolescents living with HIV (ALHIV, n=7, 12%). None of the studies based their inclusion criteria on the WHO definition of adolescence,^10^,^19^ although 20 interventions (33%) included participants across (nearly) the full range of adolescence (eg, 10–18; 9–19 years). Younger adolescents (15 years or below) were included in 92% of interventions; older adolescents (aged 16 or above) were included in 66% of interventions. Generally, both sexes were included; five interventions focused exclusively on females, one intervention targeted males only.

### Intervention types

**Figure 2:**
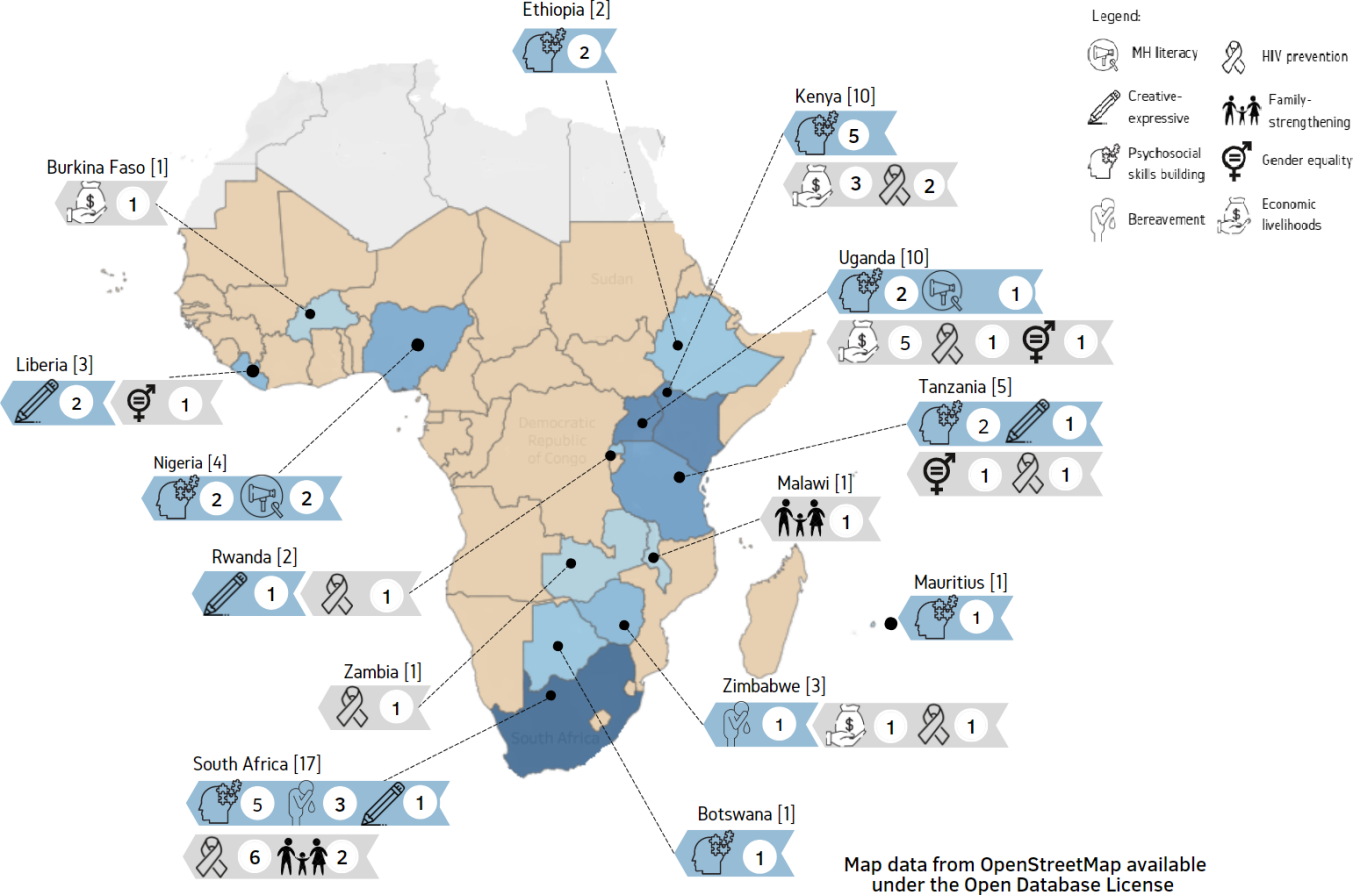
Map of type and number of interventions by country

All included evaluation studies reported AMH outcomes, yet interventions varied in the extent to which they included psychosocial programming or aimed to address AMH as a primary aim. Theoretical underpinnings of interventions were not always clearly described, therefore, the decision was made to categorise interventions based on their core focus (figure 2, table 2), which fell within the psychosocial or structural domain.

#### Psychosocial interventions

Psychosocial interventions (table 2) all had improving AMH as their primary aim. Interventions focused on MH literacy; expression and/or strengthening individual psychosocial skills.

#### Mental health literacy

Three universal MH literacy programmes were identified. Two of these used educational sessions to target adolescents in a school setting^32 33^; the third was a global WHO community awareness campaign.^34^ Quasi-experimental evaluations of these programmes found positive changes in AMH knowledge and understanding, but not in attitudes to AMH.

#### Expressive-creative interventions

Expressive-creative modalities were at the core of five interventions that aimed to support participants in expressing their thoughts and emotions in the context of bereavement. Targeted at Ebola-affected adolescents, the ‘Playing to Live’ intervention^35^ combined art, play and yoga therapy with life-skills training, while the ‘Comfort for Kids’ intervention^36^ used drawing and writing. Both interventions showed positive outcomes in pre–post evaluation studies. Targeted at orphans, a pilot RCT with orphans found that Memory therapy^37 38^—where participants explore their life story through artwork—strongly reduced general MH symptoms, whereas another small RCT found that the use of Emotional writing was not effective.^39^

#### Psychosocial skills-based interventions

Psychosocial skills development was at the core of 25 interventions. Thirteen of these shared a similar aim of equipping adolescents with psychosocial knowledge and skills to reduce distress and increase resilience in a general context of adversity. Combining elements of CBT, social emotional learning, positive psychotherapy and stress management approaches, they varied slightly in their focus to strengthen intrapersonal (eg, recognising and managing emotions, self-esteem, resilience), interpersonal (eg, communication, empathy), cognitive skills (eg, decision-making, problem-solving) and/or relaxation or mindfulness techniques. While most of these interventions aimed to bring about individual-level changes, they were mostly delivered in a group setting, using multiple workshops or therapy sessions. There was also one single-session intervention, focused on character strengths, which was delivered digitally.^40^ A large RCT (n=1288) to test this intervention in a face-to-face, multisession format is currently underway.^41^ Quantitative evaluations of these interventions (including six RCTs) reported mixed findings across a variety of outcomes. Six interventions were universal, school-based programmes,^42^,^47^ of which the ‘ERASE stress-prosocial programme’, ‘Youth First’, and the ‘Living Well Programme’ demonstrated the most consistent evidence of feasibility and effectiveness. Five interventions targeted orphans^47^,^54^ and reported predominantly positive outcomes. Three of these interventions combined therapeutic approaches with peer support modalities. For example, over the last 20 years, the Balekane EARTH programme in Botswana (previously named Ark for Children) has sent groups of orphans to a wilderness-based psychosocial strengthening camp, to build feelings of trust and kinship as a protective factor for mental health.^53^

Furthermore, an intervention aimed at specifically preventing aggression problems in young males showed positive results,^55^ while an RCT of an anxiety prevention intervention in children with visual impairments^56^ found no improvements.

Four interventions were implemented primarily in the context of bereavement, targeting orphans,^57 58^ ALHIV^59^ or adolescent females.^60^ These interventions aimed to support adolescents in expressing loss-related emotions and increase their understanding of grief and support services, in addition to equipping them with psychosocial skills. Evidence for these interventions was limited, as it consisted of two qualitative evaluations and two RCTs, of which one—for a bereavement support group called ‘Abangane’—reported small improvements.^60^

A substantial number of interventions were implemented in the context of HIV prevention and health promotion. Four of these interventions had mental health as their primary focus, with core components based on psychological therapies. A universal, community-based art programme ‘Make a Difference about Art’,^61^ where children create ‘hero’ books about their lives; a counselling intervention targeted at migrants^62^; a mobile-based, counselling and peer-support Whatsapp group for ALHIV^63^; and Sauti Ya Vijana, which combined several therapeutic approaches targeted at ALHIV,^64 65^ all aimed to reduce psychosocial problems and increase HIV insight as a mechanism to improve HIV-related outcomes, such as adherence to antiretroviral medication. None of these studies found significant changes in mental health outcomes before and after intervention. Two adolescent-centred health promotion interventions could also be classed under this category, although they had a broader focus of developing psychosocial skills and informational resources to enable adolescents to become effective HIV agents: the Young Citizens Programme increased self-efficacy and collective efficacy through public education and community mobilisation,^66^ while a peer-led education programme (‘Vhutshilo’), found no changes.^67^

### Structural interventions

Structural interventions (table 3) employed socioecological approaches to address risk factors that could lead to poor (mental) health. The psychological content of most of these interventions was limited.

**Table 3:** Included structural interventions and core components

			Psychosocial components				Structural components			
**Intervention name**	**Evaluation type and country**	**Target group and setting (if universal**)	**Creative expressive**	**MHliteracy**	**DidacticSEL/CBT**	**Bereavement/ trauma**	**HIV prevention/SRHR**	**Family strength/caregiver**	**Economic livelihood**	**IPV/gender equality**
Structural interventions										
HIV prevention and management										
CHAMP-SA^69 70^	RCT,South Africa	Universalcommunity based					C	x		
READY^68^	RCT,Kenya	Universalcommunity based			x		x	C	x	
IMARA-SA^73^	RCT,South Africa	Universal (female only)community based	x	x	x		C	x		x
VUKA^71 104^	RCT,South Africa	ALHIV			x	C	C	x		
Zvandiri (CATS)^80^	RCT,Zimbabwe	ALHIV					C			
HADITHI^81^	RCT,Kenya	ALHIV					C			
ZAMFAM^72^	Prospective cohort,Zambia	OVC/ALHIV					C	x	x	
Bantwana initiative^79^	Pre–post,Uganda	OVC			X	X	C		X	
Let’s Talk^74 105^	Pre–post,South Africa	OVC			x		C	x		
FSI-HIV^23 75 76 123^	RCT,Rwanda	Adolescent w/HIV+parent		x	X		x	C		
n/a: home visits+counselling^78^	Pre–post,South Africa	Adolescent w/HIV+parent			X		C			
Our family, our future^77 106^	RCT,South Africa	Subthreshold depression		x	x		C	x		x
Parenting interventions										
Sinovuyo Teen^85^	RCT,South Africa	Adolescents w/family conflicts			X			C	x	
n/a: adolescent parenting^84^	Waitlist control,South Africa	Adolescent parents					X	C	x	x
n/a: Early childhood education^82 83^	Waitlist control,Malawi	Adolescent mothers			x		X	C		
Gender equity and norms										
Teenage Mothers Project^86 124^	Qualitative, Uganda	Adolescent mothers			x		x	x	x	C
Girl empower^88^	RCT,Liberia	Adolescent females		x			x	x	x	C
Discover^125^	Qualitative, Tanzania	Universalschool based	x		x					C
Economic livelihood programmes										
Suubi^90 94^	RCT, Uganda	OVC							C	
Suubi-Maka^89 92 93^	RCT, Uganda	OVC							C	
Suubi+Adherence^96^	RCT, Uganda	OVC							C	
Bridges to the future^91 95 97^	RCT, Uganda	OVC							C	
Suubi4Her^98^	Protocol, Uganda	Adolescent females					x	x	C	
SHAZ!^99^	RCT, Zimbabwe	OVC					x		C	
Trickle Up Plus^100^	RCT,Burkina Faso	Ultra-poor							C	
CT-OVC^101^	RCT, Kenya	OVC							C	
OSCAR^102^	RCT, Kenya	OVC							C	
n/a: School support^103^	RCT, Kenya	OVC							C	

ALHIVadolescents living with HIVCcore intervention componentIPVintimate partner violenceMHmental healthn/ano intervention nameOVCorphans and vulnerable childrenRCTrandomised controlled trialSELsocial emotional learningSRHRsexual and reproductive health and rightsxintervention component

#### HIV prevention and management

Seven interventions were multicomponent, family-based HIV prevention programmes. Programmes were either universal,^68^,^70^ or targeted ALHIV,^71 72^ adolescent females,^73^ OVC,^74^ those with a HIV positive parent^75 76^ or mild depressive symptoms.^77^ Educational components covered topics such as caregiver relationships and family strengths; HIV transmission and treatment knowledge; talking about sensitive topics such as puberty and HIV; stress management and coping; and gender/economic empowerment. These interventions were evaluated rigorously, with six RCTs, but evidence of their impact as mental health prevention interventions was inconclusive: four interventions (‘Our Family, Our Future’, ‘FSI-HIV’, ‘IMARA-SA’ and ‘Let’s Talk’) reported improved outcomes while three others did not.

Four interventions focused on improving health service delivery processes to achieve better health outcomes in vulnerable families^78 79^ and ALHIV.^72 80^ These interventions had no didactic element, but instead provided holistic, integrated support by offering home visits, linkages to external providers and livelihood support (in two interventions). Again, the evidence was mixed: two of these interventions, the ‘Bantwana initiative’ and ‘Zvandiri’ found a positive change in MH outcomes, but the other two did not. Finally, HADITHI aimed to increase HIV status disclosure and promote resilience,^81^ but an RCT found no MH improvements.

#### Parenting interventions

While improved family connections were addressed as a protective factor in most HIV prevention interventions described above, a further three interventions aimed to strengthen parenting skills as a protective factor for (mental) health problems. Two of these programmes aimed to support early development of children,^82 83^ and prevent child abuse.^84^ The third intervention, Sinovuyo Teen^85^, targeted adolescents and their parents with sessions around stress/anger management, family problem-solving and relationship building. None of these interventions showed improvements in psychosocial outcomes.

#### Gender equity

Three interventions specifically focused on empowering women and challenging gender norms. One of these was a community-based empowerment intervention for teenage mothers, which aimed to improve their mental health by creating a supportive social environment.^86^ The two other interventions aimed to build positive mental health as a mechanism to either promote gender equity and transform gender norms,^87^ or to equip adolescent females with the skills to make strategic life choices and stay safe from sexual abuse.^88^ Robust quantitative evidence of impact has not been found: an RCT of the latter intervention found no improvements and the first two interventions were evaluated qualitatively.

#### Economic livelihood interventions

The final category consisted of 10 multiyear economic livelihood interventions. These interventions did not have any psychological content, but used asset-based approaches to promote mental health.

Five interventions in this category were led by the same principal investigator and implemented in Uganda. In Bridges to the Future, Suubi, Suubi-Maka (targeting AIDS orphans) and Suubi+adherence (targeting ALHIV), adolescents were provided with a matched savings account, in addition to financial management training and (peer) mentorship.^89^,^97^ The Suubi+Adherence intervention also incorporated HIV prevention elements of the VUKA curriculum (discussed above) to target HIV-related outcomes. An evaluation of Suubi4Her^98^, which targets females and combines this savings-led economic empowerment approach with a family strengthening component, is currently underway. Furthermore, targeting orphans, *SH*AZ! provided a vocational training package that included a conditional stipend.^99^ Trickle Up Plus, OSCAR and CT-OVC also used cash transfers, but these were given to caregivers and not directly to adolescents.^100^,^102^ A final intervention covered adolescents’ school fees and uniforms.^103^ Evidence of possible effectiveness of interventions was strongest in this category. All implemented interventions were evaluated through one or more (cluster) RCTs that that used follow-up measurements over multiple years and showed largely positive results.

### Cultural sensitivity

The following section explores how interventions addressed cultural sensitivity in original design or adaptation of their training manuals. Twenty-four interventions were said to be developed locally, often based on evidence-based models in literature. Seventeen interventions, of which 11 were developed in an HIC, were adapted from a named, pre-existing intervention. For seven interventions, the development or origin of the intervention was not described. The remaining economic livelihood interventions or those that used a service delivery approach did not require an adapted manual as such.

To different extents, 23 studies provided examples of cultural tailoring, as follows:

#### Surface structures

Most cultural tailoring took place in the ‘superficial’ surface-structure domain. Examples of this were found in 17 interventions and included the use of local illustrations/cartoons as opposed to written material in a context of low literacy^36 58 59 67 69 75 104 105^; using indigenous music, folktales/stories, metaphors, proverbs, local legends, games, crafts and/or clothing^4548 51 53^,^55 59 60 75 87 104 105^; or organising community events involving traditional leaders and ceremonies.^45 53 54 87 88^ Nigerian researchers who implemented the ‘Thinking Group’ for adolescent boys with aggression problems, adapted from the ‘BrainPower programme’ (USA), provided a specific example: in their original intervention, a principle ‘STOP, THINK before ACTING’ was explained with an analogy of traffic lights (red for Stop, amber for Think and green for Act). This was replaced with a reference to football, a game popular among many boys in the country. The referee would STOP the game for a foul, hand out a yellow card (THINK), and the player would subsequently be ACTING properly to avoid a red card and eviction from the game.^55^

#### Deep structures

Cultural tailoring at a deep structure level requires an understanding of how sociocultural forces influence mental health, and how the target population understands the cause, course and treatment of mental health issues. Authors of 13 studies described how an understanding of these ‘deep structures’ shaped their intervention, as follows:

For seven interventions, authors explained how their approach aligned with context-specific views and values: Formative work for ‘FSI-HIV’ and ‘Our Family, Our Future’, identified family communication skills, connectedness and good parenting as context-specific strategies that could promote AMH. This led authors to implement a family-based intervention focused on strengthening parent–child relationships and family narrative.^23 106^ Similarly, authors stated that since SSA societies are often highly collectivistic, group approaches that promote family, mutual unity and collective responsibility, would be most appropriate.^54^ They highlight that interventions should not over-emphasise Western individualistic values such as independence and critical thinking that might be at odds with local values.^45^ Strength-based approaches that use conceptualisations of positive mental health rather than psychopathology were also said to be more suitable when a focus on psychopathology might invoke stigma.^2340^,^42^

In terms of context-specific content or focus of interventions, it could be argued that sessions on topics like HIV and orphanhood respond to risk factors for mental health issues that are particularly relevant in SSA contexts. However, specific descriptions of content adaptations to reflect the local context were few: CHAMP-SA and VUKA both had an increased focus on loss and bereavement, as it was felt that this set the tone for all further communications in the intervention.^69 104^ The cartoon used in the intervention was adapted to include a character who kept a Memory Box, to encourage participants to create one themselves. Similarly, the cartoon in Let’s Talk included a character who was dealing with bereavement and chronic illness, while living in foster care.^105^ A bereavement intervention was adapted so that it reflected a reality of multiple losses, as opposed to one loss. In addition, as many participants lived in a situation of ongoing adversity, coping techniques focused on dealing with challenges that cannot be changed and are out of one’s control.^59^ In Rwanda, the FSI-HIV intervention included an optional session on genocide-related trauma psychoeducation.^23^

Explorations into local understanding, terminologies or expressions of mental health were described as part of developmental work for five interventions: In the adaptation phase of their CBT for South African children with visual impairments, authors explored understanding of the concepts ‘feelings’ and ‘thoughts’. Although participants were found to have an adequate understanding of these concepts through previous exposure within the school curriculum, the authors stressed the importance of such explorations preintervention.^107^ Formative work for the ERASE intervention described that Tanzanian children had not had this exposure, and therefore, this intervention allowed more time to learn about relevant concepts. In addition, body-oriented techniques were added, since distress was often expressed through somatic complaints.^45^ Explorations of local conceptualisations and terminologies of mental health issues were specified by some: exploratory work for the FSI-HIV^23^ identified six locally defined syndromes indicative of mental health needs of children, while others used local terminology such as ‘stress’ and ‘everything is too much’ to refer to depression.^58 106^

Finally, four groups of authors described how their intervention integrated local traditions and interpretations regarding the causes and treatment of AMH issues: Instead of employing a Western cognitive-behavioural framework for dealing with ‘automatic negative thoughts’ that was part of the original *ERASE* manual, the adaptation incorporated a local perspective that negative thoughts represent admonitions from ancestors for misdeeds. Students were encouraged to reflect on their conduct and learn from it rather than challenge or dispute negative thoughts. Similarly, in addition to Western desensitisation strategies, students were introduced to traditional ceremonial healing. A collective grief ritual was also performed during a session.^45^ The Abangane bereavement intervention also merged CBT sessions with discussions around local traditions and myths about death and grief.^60^ The Balekane EARTH intervention is a wilderness-based retreat which follows rites of passage that shared many similarities to historical initiation as part of Setswana culture in Botswana. In addition, ceremonies are held when children depart and return from the camp.^54^ The formative work for *VUKA* showed that in the South African context, interventions need to address social interpretations of mental ill health such as bewitchment and demonisation, yet the authors did not describe how VUKA did this.^71^

### The evidence base: systematic review

This section analyses findings from 39 RCTs, which measured different combinations of outcomes related to positive mental health or symptoms of mental disorders. Most studies used relatively short follow-up times, with few measuring outcomes more than 1 year after intervention. Online supplemental file 2 provides an overview of risk-of-bias assessments, which found reason for ‘some concern’ or ‘high risk of bias’ in more than half of all RCTs. Table 4 provides an at-a-glance, colour-coded overview to accompany the following summary:

The effectiveness of 12 psychosocial interventions was explored with RCTs. Most had small sample sizes, ranging from 46 to 613. Mixed results across a range of MH measurements were reported: five found positive changes across all measures, three reported partial impact and three found no improvements. Nine HIV prevention/management interventions were evaluated by RCTs, which also often used limited sample sizes ranging from 60 to 557. One study found improvements on all measurements of mental health, while three found partial improvements and five found no changes. RCTs further evaluated nine economic livelihood interventions, with larger sample sizes ranging from 286 to 1960. Three studies found partial improvements, while six studies reported positive changes across all mental health measurements used. Finally, RCTs of a parenting intervention (n=553)^85^ and a gender equity intervention (n=1159),^88^ which both had a livelihoods component, did not report any changes. No patterns emerged in terms of superior effectiveness depending on target population and intervention duration.

**Table 4:** Overview of RCT outcomes

(Cluster) RCT			Outcome measure+effect size at latest follow-up (if applicable)							
**Author, country**	**Intervention name (duration**)	**Bias, sample size, F: follow-up times**	**General MH symptoms**	**Depression**	**Anxiety**	**Hopeless ness**	**Self-evaluation**	**Resilience**	**Trauma**	**Other**
Psychosocial interventions										
Harding	Memory therapy5 days	High risk,N=46, F=6 weeks	BSI: d=0.80SDQ: d=0.76				SES: d=1.33SEQC: d=1.26			
Unterhitzen-berger and Rosen	Emotional writing3 weeks (1/week)	Some concern,N=69, F=n/a		MINI-KID						PGQ-A (grief)
Berger, Tanzania	ERASE-stress-prosocial8 weeks	Some concern,N=183F=8 mths	SDQ: Hyperactive:Partial η^2^=0.24		SCAS:Partial η^2^=0.25					SDQ: - Social difficulties: Partial η^2^=0.20. -Prosocial:Partial η^2^=0.22
De Villiers and vd Berg, South Africa	Resiliency programme15 sessions	Low risk,N=161, F=3 mths					FORQ-self-appraisal: d=0.53	RSCA		BERS: interpersonal/intra personal strenghts
Rivet-Duval, Mauritius	RAP-A programme11 weeks (1/week)	High risk,N=160, F=6 mths		RADS2:T1 Reduced		BHS: T1 increased	SES:d=0.46	YCI:d=0.27		
Mutiso, Kenya	Psychoeducation and life skills, 4 days	Low risk, N=630F=3, 6, 9 mths	YSR:d np							
Kumakech, Uganda	Peer support group10 weeks (2/week)	High risk, N=326F=n/a		BYI:d=0.84	BYI:d=0.24		TSCS (self-concept)			BYI-Anger:*d=*0.68
Osborn, Kenya	Shamiri digital1 session	Some concern, N=103, F=n/a	WEMWBS	PHQ-9: d=*0.50*	GAD-7					
Visagie, South Africa	PAM programme5 weeks (2/week)	Some concern,N=52, F=3 mths		RCAD	RCAD					PSWQ (worry)
Thurman, South Africa	Abangane8 weeks (1/week)	Low,N=453, F=3 mths	BPM-PFd=*0.31*	CESD-Cd=0.21						Grief: CBI-G: n.s. IGTS: d=0.21; ICG-RC: d=0.14
Thurman, South Africa	Interpersonal group therapy 16 weeks	Some concern, N=489, F=3, 12 mths		CESD-C						
Carlson, Tanzania	Young citizens28 weeks (1/week)	Low risk,N=613, F=4 months					Self-efficacyd=0.27+0.30			Emotional control: d=0.17
HIV and MH interventions										
Dow, Tanzania	Sauti Ya Vijana10 weeks (1/week)	Low risk, N=105F=6, 12, 18 mths	SDQ	PHQ-9					UCLA Trauma	
Kuo, South Africa	Our Family our Future3 weeks (1/week)	Low risk,N=73, F=3 mths		CESDd*=*0.72				CDRS		
Bhana, South Africa	VUKA3 months (2/month)	High risk,N=65, F=2 weeks	SDQ	CDI						
Betancourt, Rwanda	FSI-HIV8 weeks	Some concern,N=170 F=3 months	Conduct problems	CESD-Cd np						
Donenberg, South Africa	IMARA-SA10 hours (1 or 2 days)	Low risk,N=60, F=6–10 months		PHQ-9d np	GAD-7d np				PC-PTSD-5	
Puffer, Kenya	READY9 sessions	Some concern, N=237, F=1, 3 mths	SDQ	CDI	MASC		SES			
Vreeman, Kenya	HADITHI	Low risk, N=285,F=6, 12, 18, 24 months	SDQ:Increased F2	PHQ-9Increased F2						
Willis, Zimbabwe	Zvandiri (CATS)Service delivery	Low risk,N=88,F=12 months					Confidence, self-esteem, self-worth: d np			
Bell, South Africa	CHAMP-SA10 weeks (1/week)	High risk,N=557, F=unknown	GHQCPBC		CMAS					Psychological autonomy
Economic livelihood programmes										
Dufour	*SHAZ!*6 months	Low risk, N=315F=12, 18, 24 months	SSQ: Only at T4d=0.63							
Shangani	OSCAR	Some concern,N=655, F=36 months		CDI	CMASd=0.31	Pos outlook: d=0.22			PTSD:d=0.38	
Han, Ssewemala Karimli	Suubi-Maka12 months	High risk/low risk,N=317, F=24 months		CDId np		BHSd np	TSCSd np			
Ismayilova	Trickle Up Plus24 months	Some concern, N=318, F=12, 24 months		CESD*d=*0.39			SES		CRIESOnly at T1	
Ssewemala x2 Kivumbi	Bridges to the future24 months	Low/high, N=1383, F=12, 24, 36, 48 months		CDIOnly at F1/2		BHSd np	TSCS, SESiinp			
Ssewemala 2009, Ssewemala 2012	Suubi12 months	Low risk, N=286F=10, 20 months		CDId=0.65			TSCS Only at F1d=0.43			
Cavazos-Rehg	Suubi+adherence24 months	High risk, N=702, F=12, 24, 36, 48 months		CDIOnly at T2		BHSOnly at T2	TSCS			
Green	School support programme	High risk, N=835F=12, 24, 36, 48 months		CESDd=*0.28*						
Kilburn	CT-OVC	Some concern, N=1960, F=24, 48 months		CESD Male only d=0.38		Hope: Male only d=*0.26*				
Other structural interventions										
Cluver, South Africa	Sinovuyo Teen14 weeks	N=553F=5–9 months		MINI-KIDCDI						CBC (aggression+rule breaking)
Ozler, Liberia	Girl Empower, 39 weeks	N=1159, F=24 months	SMFQ				SES		CRIES	

BERS-2Behavioural and Emotional Rating ScaleBHSBeck’s Hopelessness ScaleBPM-PFBrief Problem Monitor-Parent formBSIBrief symptom inventoryBYIBeck’s Youth InventoryCBCChild Behaviour ChecklistCBICore Bereavement ItemsCDIchild depression inventoryCDRSConnor-Davidson Resilience ScaleCESDCentre for Epidemiological Studies Depression ScaleCMASChildren’s Manifest Anxiety ScaleCPBCChild Problem Behaviour ChecklisCRIESChildren’s Revised impact of eventsFORQFortitude QuestionnaireGAD-7Generalised Anxiety DisorderGHQGeneral Health QuestionnaireICG-RCInventory of Complicated GriefIGTSIntrusive Grief Thoughts ScaleMASCMulti-Dimensional Anxiety Scale for ChildrenMINI-KIDMini International Neuropsychiatric InterviewOVCorphans/vulnerable childrenPC-PTSD-5Primary Care Post-Traumatic Stress Disorder ScreenPGQ-AProlonged Grief Questionnaire for AdolescentsPHQ-9Patient Health QuestionnaireRADS2Reynolds Adolescent Depression scaleRCADRevised Children’s Anxiety and Depression ScaleRCTrandomised controlled trialRSCAResiliency ScaleSCASSpence Children’s Anxiety ScaleSDQStrengths and Difficulties QuestionnaireSEQCSelf-Efficacy Questionnaire for ChildrenSESRosenberg Self-Esteem ScaleSMFQShort Mood and Feelings QuestionnaireSSQShona Symptom QuestionnaireTSCSTennessee Self-Concept ScaleUCLA TraumaUCLA Post Traumatic Stress Symptoms Exposure Screener and Reaction IndexWEMWBSWarwick Edinburgh Mental Wellbeing ScaleYCIYouth Coping Index YSRYouth self-report

#### Indicators of positive mental health

Studies showed varying impacts across a wide range of positive mental health indicators, which typically fall within the realm of mental health promotion:

Self-evaluation outcomes were measured by three universal psychosocial skills-based interventions, which all reported moderate improvements. The evaluation of Memory Therapy, a creative-expressive intervention, found improved self-esteem (d=1.33) and self-efficacy (d=1.25). Family-based HIV and MH prevention interventions did not typically measure these outcomes; there was one study that measured self-esteem but found no improvements. Zvandiri, an HIV service delivery intervention found improved confidence, self-esteem and self-worth, although effect size calculations were not possible. Five similar economic livelihood interventions did not find consistent improvements in self-evaluation scores: only Suubi-Maka and Bridges to the Future found improved levels of self-concept and self-esteem (effects size calculations not possible).

Interpersonal skills were measured by two universal psychosocial interventions, of which only the ERASE stress prosocial intervention reported substantial improvements in social difficulties and prosocial skills. Hopelessness was assessed in one universal psychosocial intervention, which found no improvements, and across five similar economic livelihood interventions targeted at OVCs, which all reported reduced hopelessness levels, with small or unknown effect sizes. Two psychosocial and one family-based HIV and MH intervention assessed resilience. Only the universal, school-based *RA*P-A programme reported a small improvement (d=0.27) in scores. Grief was addressed by two psychosocial interventions targeted at OVCs, of which only Abangane reported small improvements (d=0.14 and d=0.21).

#### Impact on indicators of negative mental health

In terms of indicators of negative mental health, almost all studies included a measure of general mental health symptoms, depression and/or anxiety. Of four universal psychosocial interventions, the ERASE programme reported an improvement in anxiety scores, while a psychoeducation and life skills programme generally improved internalising and externalising problems (unknown effect size). Shamiri digital moderately improved depression but did not change general well-being or anxiety. Of five psychosocial/bereavement interventions targeted at OVCs, three reported significant changes: Memory Therapy (d=0.80), a peer support group (d=0.84) and Abangane (d=0.21). Of the seven family-based HIV and MH prevention interventions that measured MH symptomology, only three reported improvements in depression/anxiety scores: Our Family, Our Future reported a large effect size (d=0.72), while FSI-HIV and IMARA-SA reported improvements with unknown effect sizes. HADITHI, a HIV status disclosure intervention, found increased rates of MH symptoms at 6 months postintervention, but this reduced at later follow-ups. Economic livelihood interventions showed a more consistent impact, with reduced symptoms of MH conditions reported across all studies. A moderate effect size was reported for the Suubi intervention (d=0.65), while others reported small or unknown effect sizes. However, reductions were not all sustained at follow-ups of more than 2 years,^94^,^96^ while SHAZ! only reported a moderate effect size improvement after 24 months (d=0.63). The CT-OVC intervention only showed small improvements (d=0.38) in scores of males.^101^

Trauma was assessed by four studies. Two MH and HIV prevention interventions (Sauti Ya Vijana and IMARA-SA) did not find improvements in trauma scores, but two economic livelihood programmes with OVCs (OSCAR and Trickle Up Plus) did.

## Discussion

The aim of this combined scoping and systematic review was to explore the scope of research into promotive/preventive AMH interventions across SSA and to gain insight into effectiveness and cultural tailoring of these interventions. A total of 79 papers were identified, related to 61 primary interventions. Although research into the topic is emerging with most papers published after 2015, the identified evidence was relatively limited in terms of target populations and geographical spread, with three countries (South Africa, Kenya or Uganda) accounting for 60% of the research. However, in terms of intervention aims, approaches and outcomes, the scope of research was broad. This was expected and reflects many possible pathways to promoting mental health and preventing mental illness either through psychosocial or structural interventions.

In addition to three mental health literacy interventions, this review found 30 psychosocial interventions, often targeting individual-level outcomes. Interventions had core components linked to creative-expressive approaches, cognitive–behavioural/socioemotional skills building and bereavement. Five of these were universal, school-based interventions, while most were targeted interventions, focused on orphans or HIV positive adolescents. Psychosocial skills-based components of these interventions appear similar to those reported in previous reviews.^10 13 20^ Included interventions had the primary aim of promoting mental health as the end goal, or as a clearly specified stop along the road to reducing risky behaviours and/or promoting physical health. Others were mainly intended to achieve another aim, but also measured mental health outcomes. This was particularly the case for the remaining 28 structural interventions, which included HIV prevention/management interventions; parenting/family strengthening interventions; economic livelihood interventions and gender equality interventions.

As also reported in previous reviews,^17 19 20^ heterogeneity in terms of programme content, delivery, duration and study sample made it difficult to draw general conclusions about the effectiveness of interventions as a whole. RCTs of three universal, school-based interventions (ERASE, RAP-A and a psychoeducational life skills programme^44 50 108^) showed promising results. The effectiveness of these skills-based interventions has been established in HICs^108 109^ and these studies now provide initial evidence of effective cross-cultural transferability of such approaches to SSA contexts. Indications of superior effectiveness for certain psychosocial interventions in certain target populations were not found. However, in the context of bereavement, memory therapy and a peer-support group for orphans^38 52^ were found to be effective. While a review of promotion interventions in LMICs^20^ concluded that multicomponent community-based interventions showed a positive impact, the current review adds further nuance to this conclusion. Namely, the strongest evidence of effectiveness came from economic livelihood interventions. Many RCTs of other structural, community-based interventions, such as HIV and mental health prevention interventions, showed mixed results. This does not necessarily mean that these interventions were not successful, as many measured symptom levels -often below clinical levels at the start of the intervention—as opposed to well-being indicators. Furthermore, the relatively short follow-up times of these interventions might not have been long enough to determine effect on diagnosable disorders. More high-quality, longitudinal studies that use appropriate analytical approaches are needed to establish the impact of prevention interventions on the onset of mental health disorders.

To gain the full picture on AMH prevention interventions in SSA, the current review should be read alongside a recent review on substance use prevention interventions, which found evidence of effectiveness for individual-focused interventions, rather than school-based approaches.^110^ This review also reported a limited geographical spread of studies across the region and includes an interesting discussion around the dominance of South-African research that we feel is also relevant for the current review.

Examples of cultural tailoring were found in 23 interventions, yet this was often at the surface level. At deep structure level, few interventions specified if and how they incorporated African worldviews and contexts (eg, related to spiritual beliefs). One of the interventions with the most contextualisation (ERASE) has led to good results, but further research is needed to understand how this impacts intervention effectiveness and/or accuracy of measurements. It should be noted, however, that few authors published details of the full intervention adaptation process from development to evaluation. This may mean that considerations of local context have been missed. The lack of clear reporting on adaptations made, could also be a reflection of the lack of a standardised, evidence-based framework for cultural adaptation.^111^

This review highlighted that most interventions were delivered in the context of HIV, either by targeting (AIDS) orphans, HIV positive adolescents, embedding HIV prevention approaches, or by framing poor mental health as a risk factor for risky sexual behaviour. While this shows a general responsiveness of interventions to the SSA context, it is important to recognise that many adolescents in SSA face significant daily adversities,^8^ regardless of their HIV or orphanhood status. All adolescents would reap the benefits of being mentally well. Universal, school-based approaches that foster multisectoral action would allow for a wide reach of generic mental health promotion and prevention activities. These are strongly recommended by the WHO since they are considered relatively easy to implement and less likely to cause stigmatisation compared with interventions that require screening.^4^ Yet, this review showed that there have so far been limited attempts to develop or adapt these in SSA. Teachers are often considered to be best placed to deliver school-based interventions, but their ability to do so may be inhibited by stigma and limited MH literacy.^112^ These approaches would, therefore, need to include adolescent-targeted as well as teacher-targeted components. For the latter, a potential resource might be the WHO manual for mental health in schools, aimed at building MH literacy in educators in resource-limited settings.^113^ Still, teacher-dependent interventions need to consider the potential burden on teachers who are often responsible for a large number of children and may have few opportunities for professional development in the face of challenges of teaching in a low-resource context.^114^ This could signal the need for non-teaching professionals to deliver school-based MH programmes.^115^

A major limitation of using school-based approaches for AMH promotion/prevention is that this would not reach adolescents outside of the school system. This specific population often includes a higher proportion of adolescents with increased vulnerability to MH conditions, such as orphans, those in informal settlements and street youth.^114^ While this review identified targeted interventions at individual or interpersonal/family level that could perhaps be scaled up to include a broader range of adolescents, it did not identify any universal interventions at community level (such as the Communities That Care approach^116^) aimed at forming community coalitions to prevent negative MH outcomes. Such interventions should also include specific stigma-reduction components, to combat the far-reaching negative impact of mental health-related stigma—present in many SSA communities—on health seeking behaviour and social inclusion.^117^

In terms of further research gaps, there were no studies that explored opportunities for national or regional scale-up and sustainability of promising interventions. Most economic livelihood studies built on the work of the same principal investigator in Uganda. Explorations of this work in other SSA settings should explore transferability of findings. There were also no studies that included elements related to suicide prevention, lifestyle approaches around exercise/nutrition and social media. Such components would make interventions responsive to contemporary developments in the region, including high rates of suicidal ideation and increasing obesity among adolescents.^118 119^

While we believe the rigorous and transparent design and the lack of language restrictions were significant strengths of this review, it also had some limitations. First, only interventions that were delivered to adolescents and that measured AMH outcomes were included. Because of this, some interventions that could be classed as AMH promotion interventions (eg, housing interventions, teacher-training) were not considered. Still, this review applied broad inclusion criteria, which led to the inclusion of a large number of heterogeneous interventions. As a result, it was felt that a meta-analysis was not possible, and the decision was made to focus on intervention types, rather than on psychosocial skills-based components to organise findings. Such a component-based approach might have allowed us to gain deeper insight into whether certain psychosocial exercises (eg, CBT vs mindfulness-based activities) showed superior effectiveness over others, as was explored by Skeen *et al*.^10^ Finally, this review did not include grey literature, which means we may have missed interventions delivered by implementers that do not have the capacity to publish.

## Conclusion

This review showed that the scope of research into AMH promotion and prevention interventions in SSA was limited in terms of geographical spread, but broad in terms of intervention types, components and outcomes. The lives of many adolescents in SSA are characterised by socioeconomic deprivation, family disruption and poor physical health. This, and an awareness of the intimate connection between mental health, physical health and (risky) behaviour, has clearly shaped research into AMH interventions in the region. There were few universal, school-based interventions that aimed to improve psychosocial skills of all adolescents. Instead, many interventions were targeted at orphans or HIV patients. Around half of the interventions were structural, and addressed HIV, parenting, gender equity and income. Although they measured AMH outcomes, they did not necessarily contain psychosocial programming. Evidence for psychosocial and HIV prevention interventions was mixed; economic livelihood interventions appeared to have the most consistent evidence of effectiveness. While some interesting examples of cultural tailoring were found, future studies could do more to consider and/or report this.

## supplementary material

10.1136/bmjph-2023-000037 online supplemental file 1

10.1136/bmjph-2023-000037 online supplemental file 2

## Data Availability

All data relevant to the study are included in the article or uploaded as online supplemental information.
