# Supplementary material for: Promotive and preventive interventions for adolescent mental health in Sub-Saharan Africa: a combined scoping and systematic review
Source: BMJ Public Health. 2023 Dec 5;1(1):e000037. doi: 10.1136/bmjph-2023-000037 (PMC11812691; doi:10.1136/bmjph-2023-000037)
Supplement: online supplemental file 1 [file bmjph-1-1-s001.pdf]

MEDLINE search

#	Query	Limiters/Expanders
**S21**	S5 AND S10 AND S15 AND S20	Limiters - Date of Publication: 20000101-<br>Expanders - Apply equivalent subjects<br>Search modes - Boolean/Phrase
**S20**	S16 OR S19	Expanders - Apply equivalent subjects<br>Search modes - Boolean/Phrase
**S19**	S17 OR S18	Expanders - Apply equivalent subjects<br>Search modes - Boolean/Phrase
**S18**	AB "sub-saharan Africa" OR AB Angola OR AB Benin OR AB Botswana OR AB "Burkina Faso" OR AB Burundi OR AB Cameroon OR AB "Cape Verde" OR AB "Central African Republic" OR AB Chad OR AB Comoros OR AB "Republic of the Congo" OR AB "Democratic Republic of the Congo" OR AB "Cote d'Ivoire" OR AB "Ivory Coast" OR AB Djibouti OR AB "Equatorial Guinea" OR AB Eritrea OR AB Eswatini OR AB Ethiopia OR AB Gabon OR AB "The Gambia" OR AB Ghana OR AB Guinea OR AB "Guinea-Bissau" OR AB Kenya OR AB Lesotho OR AB Liberia OR AB Madagascar OR AB Malawi OR AB Mali OR AB Mauritania OR AB Mauritius OR AB Mozambique OR AB Namibia OR AB Niger OR AB Nigeria OR AB Rwanda OR AB "Sao Tome and Principe" OR AB Senegal OR AB Seychelles OR AB "Sierra Leone" OR AB "South Africa" OR AB "South Sudan" OR AB Sudan OR AB Swaziland OR AB Tanzania OR AB Togo OR AB Uganda OR AB Zambia OR AB Zimbabwe	Expanders - Apply equivalent subjects<br>Search modes - Boolean/Phrase
**S17**	TI "sub-saharan Africa" OR TI Angola OR TI Benin OR TI Botswana OR TI "Burkina Faso" OR TI Burundi OR TI Cameroon OR TI "Cape Verde" OR TI "Central African Republic" OR TI Chad OR TI Comoros OR TI "Republic of the Congo" OR TI "Democratic Republic of the Congo" OR TI "Cote d'Ivoire" OR TI "Ivory Coast" OR TI Djibouti OR TI "Equatorial Guinea" OR TI Eritrea OR TI Eswatini OR TI Ethiopia OR TI Gabon OR TI "The Gambia" OR TI Ghana OR TI Guinea OR TI "Guinea-Bissau" OR TI Kenya OR TI Lesotho OR TI Liberia OR TI Madagascar OR TI Malawi OR TI Mali OR TI Mauritania OR TI Mauritius OR TI Mozambique OR TI Namibia OR TI Niger OR TI Nigeria OR TI Rwanda OR TI "Sao Tome and Principe" OR TI Senegal OR TI Seychelles OR TI "Sierra Leone" OR TI "South Africa" OR TI "South Sudan" OR TI Sudan OR TI Swaziland OR TI Tanzania OR TI Togo OR TI Uganda OR TI Zambia OR TI Zimbabwe	Expanders - Apply equivalent subjects<br>Search modes - Boolean/Phrase

**S16**	(MH "Africa South of the Sahara+") OR (MH "Angola") OR (MH "Benin") OR (MH "Togo") OR (MH "Nigeria") OR (MH "Botswana") OR (MH "Namibia") OR (MH "Zimbabwe") OR (MH "Burkina Faso") OR (MH "Burundi") OR (MH "Cameroon") OR (MH "Equatorial Guinea") OR (MH "Congo") OR (MH "Central African Republic") OR (MH "Cabo Verde") OR (MH "South Africa") OR (MH "Chad") OR (MH "Niger") OR (MH "Comoros") OR (MH "Democratic Republic of the Congo") OR (MH "Djibouti") OR (MH "Somalia") OR (MH "Ethiopia") OR (MH "Guinea-Bissau") OR (MH "Eritrea") OR (MH "Eswatini") OR (MH "Gabon") OR (MH "Sao Tome and Principe") OR (MH "Gambia") OR (MH "Ghana") OR (MH "Cote d'Ivoire") OR (MH "Guinea") OR (MH "Kenya") OR (MH "Lesotho") OR (MH "Liberia") OR (MH "Sierra Leone") OR (MH "Madagascar") OR (MH "Mauritius") OR (MH "Malawi") OR (MH "Mali") OR (MH "Senegal") OR (MH "Mauritania") OR (MH "Seychelles") OR (MH "Mozambique") OR (MH "Tanzania") OR (MH "Rwanda") OR (MH "Sudan") OR (MH "South Sudan") OR (MH "Uganda") OR (MH "Zambia")	Expanders - Apply equivalent subjects<br>Search modes - Boolean/Phrase
**S15**	S11 OR S14	Expanders - Apply equivalent subjects<br>Search modes - Boolean/Phrase
**S14**	S12 OR S13	Expanders - Apply equivalent subjects<br>Search modes - Boolean/Phrase
**S13**	AB child* OR AB adolescen* OR AB teen* OR AB juvenile OR AB youth OR AB "young adult" OR AB “young person” OR AB “young people”	Expanders - Apply equivalent subjects<br>Search modes - Boolean/Phrase
**S12**	TI child* OR TI adolescen* OR TI teen* OR TI juvenile OR TI youth OR TI "young adult" OR TI “young person” OR TI “young people”	Expanders - Apply equivalent subjects<br>Search modes - Boolean/Phrase
**S11**	(MH "Adolescent") OR (MH "Child+") OR (MH "Young Adult")	Expanders - Apply equivalent subjects<br>Search modes - Boolean/Phrase
**S10**	S6 OR S9	Expanders - Apply equivalent subjects<br>Search modes - Boolean/Phrase
**S9**	S7 OR S8	Expanders - Apply equivalent subjects<br>Search modes - Boolean/Phrase
**S8**	AB promot* OR AB prevent* OR AB awareness OR AB campaign OR AB training OR AB education OR AB intervention	Expanders - Apply equivalent subjects<br>Search modes - Boolean/Phrase

**S7**	TI promot* OR TI prevent* OR TI awareness OR TI campaign OR TI training OR TI education OR TI intervention	Expanders - Apply equivalent subjects<br>Search modes - Boolean/Phrase
**S6**	(MH "Primary Prevention") OR (MH "Health Promotion+")	Expanders - Apply equivalent subjects<br>Search modes - Boolean/Phrase
**S5**	S1 OR S4	Expanders - Apply equivalent subjects<br>Search modes - Boolean/Phrase
**S4**	S2 OR S3	Expanders - Apply equivalent subjects<br>Search modes - Boolean/Phrase
**S3**	AB "mental health" OR AB "mental health literacy" OR AB psychological OR AB psychosocial OR AB wellbeing OR AB emotional OR AB coping OR AB resilien* OR AB "mental health stigma" OR AB distress	Expanders - Apply equivalent subjects<br>Search modes - Boolean/Phrase
**S2**	TI "mental health" OR TI "mental health literacy" OR TI psychological OR TI psychosocial OR TI wellbeing OR TI emotional OR TI coping OR TI resilien* OR TI "mental health stigma" OR TI distress	Expanders - Apply equivalent subjects<br>Search modes - Boolean/Phrase
**S1**	(MH "Mental Health") OR (MH "Stress, Psychological") OR (MH "Resilience, Psychological") OR (MH "Social Support")	Expanders - Apply equivalent subjects<br>Search modes - Boolean/Phrase

CINAHL search

#	Query	Limiters/Expanders
**S17**	S4 AND S8 AND S12 AND S16	Limiters - Published Date: 20000101-<br>Expanders - Apply equivalent subjects<br>Search modes - Boolean/Phrase
**S16**	S13 OR S14 OR S15	Expanders - Apply equivalent subjects<br>Search modes - Boolean/Phrase
**S15**	AB "sub-saharan Africa” OR AB Angola OR AB Benin OR AB Botswana OR AB “Burkina Faso” OR AB Burundi OR AB Cameroon OR AB “Cape Verde” OR AB “Central African Republic” OR AB Chad OR AB Comoros OR AB “Republic of the Congo” OR AB “Democratic Republic of the Congo” OR AB "Cote d'Ivoire" OR AB "Ivory Coast" OR AB Djibouti OR AB "Equatorial Guinea" OR AB Eritrea OR AB Eswatini OR AB Ethiopia OR AB Gabon OR AB “The Gambia” OR AB Ghana OR AB Guinea OR AB “Guinea-Bissau” OR AB Kenya OR AB	Expanders - Apply equivalent subjects<br>Search modes - Boolean/Phrase

	Lesotho OR AB Liberia OR AB Madagascar OR AB Malawi OR AB Mali OR AB Mauritania OR AB Mauritius OR AB Mozambique OR AB Namibia OR AB Niger OR AB Nigeria OR AB Rwanda OR AB “Sao Tome and Principe” OR AB Senegal OR AB Seychelles OR AB "Sierra Leone" OR AB “South Africa” OR AB “South Sudan” OR AB Sudan OR AB Swaziland OR AB Tanzania OR AB Togo OR AB Uganda OR AB Zambia OR AB Zimbabwe	
**S14**	TI "sub-saharan Africa” OR TI Angola OR TI Benin OR TI Botswana OR TI “Burkina Faso” OR TI Burundi OR TI Cameroon OR TI “Cape Verde” OR TI “Central African Republic” OR TI Chad OR TI Comoros OR TI “Republic of the Congo” OR TI “Democratic Republic of the Congo” OR TI "Cote d'Ivoire" OR TI "Ivory Coast" OR TI Djibouti OR TI "Equatorial Guinea" OR TI Eritrea OR TI Eswatini OR TI Ethiopia OR TI Gabon OR TI “The Gambia” OR TI Ghana OR TI Guinea OR TI “Guinea-Bissau” OR TI Kenya OR TI Lesotho OR TI Liberia OR TI Madagascar OR TI Malawi OR TI Mali OR TI Mauritania OR TI Mauritius OR TI Mozambique OR TI Namibia OR TI Niger OR TI Nigeria OR TI Rwanda OR TI “Sao Tome and Principe” OR TI Senegal OR TI Seychelles OR TI "Sierra Leone" OR TI “South Africa” OR TI “South Sudan” OR TI Sudan OR TI Swaziland OR TI Tanzania OR TI Togo OR TI Uganda OR TI Zambia OR TI Zimbabwe	Expanders - Apply equivalent subjects<br>Search modes - Boolean/Phrase
**S13**	(MH "Africa South of the Sahara+") OR (MH "Angola") OR (MH "Benin") OR (MH "Togo") OR (MH "Nigeria") OR (MH "Botswana") OR (MH "Namibia") OR (MH "Zimbabwe") OR (MH "Burkina Faso") OR (MH "Burundi") OR (MH "Cameroon") OR (MH "Equatorial Guinea") OR (MH "Congo") OR (MH "Central African Republic") OR (MH "Cape Verde") OR (MH "South Africa") OR (MH "Chad") OR (MH "Niger") OR (MH "Comoros") OR (MH "Democratic Republic of the Congo") OR (MH "Djibouti") OR (MH "Somalia") OR (MH "Ethiopia") OR (MH "Guinea-Bissau") OR (MH "Eritrea") OR (MH "Swaziland") OR (MH "Gabon") OR (MH "Gambia") OR (MH "Ghana") OR (MH "Cote d'Ivoire") OR (MH "Guinea") OR (MH "Kenya") OR (MH "Lesotho") OR (MH "Liberia") OR (MH "Sierra Leone") OR (MH "Madagascar") OR (MH "Malawi") OR (MH "Mali") OR (MH "Senegal") OR (MH "Mauritania") OR (MH "Mozambique") OR (MH "Tanzania") OR (MH "Rwanda") OR (MH "Sudan") OR (MH "South Sudan") OR (MH "Uganda") OR (MH "Zambia")	Expanders - Apply equivalent subjects<br>Search modes - Boolean/Phrase
**S12**	S9 OR S10 OR S11	Expanders - Apply equivalent subjects<br>Search modes - Boolean/Phrase
**S11**	AB promot* OR AB prevent* OR AB awareness OR AB campaign OR AB training OR AB education OR AB intervention	Expanders - Apply equivalent subjects<br>Search modes - Boolean/Phrase
**S10**	TI promot* OR TI prevent* OR TI awareness OR TI campaign OR TI training OR TI education OR TI intervention	Expanders - Apply equivalent subjects

		Search modes - Boolean/Phrase
**S9**	(MH "Health Promotion+")	Expanders - Apply equivalent subjects<br>Search modes - Boolean/Phrase
**S8**	S5 OR S6 OR S7	Expanders - Apply equivalent subjects<br>Search modes - Boolean/Phrase
**S7**	AB "mental health" OR AB "mental health literacy" OR AB psychological OR AB psychosocial OR AB wellbeing OR AB emotional OR AB coping OR AB resilien* OR AB "mental health stigma" OR AB distress	Expanders - Apply equivalent subjects<br>Search modes - Boolean/Phrase
**S6**	TI "mental health" OR TI "mental health literacy" OR TI psychological OR TI psychosocial OR TI wellbeing OR TI emotional OR TI coping OR TI resilien* OR TI "mental health stigma" OR TI distress	Expanders - Apply equivalent subjects<br>Search modes - Boolean/Phrase
**S5**	(MH "Mental Health") OR (MH "Stress, Psychological+") OR (MH "Support, Psychosocial+")	Expanders - Apply equivalent subjects<br>Search modes - Boolean/Phrase
**S4**	S1 OR S2 OR S3	Expanders - Apply equivalent subjects<br>Search modes - Boolean/Phrase
**S3**	AB child* OR AB adolescen* OR AB teen* OR AB juvenile OR AB youth OR AB "young adult" OR AB “young person” OR AB “young people”	Expanders - Apply equivalent subjects<br>Search modes - Boolean/Phrase
**S2**	TI child* OR TI adolescen* OR TI teen* OR TI juvenile OR TI youth OR TI "young adult" OR TI “young person” OR TI “young people”	Expanders - Apply equivalent subjects<br>Search modes - Boolean/Phrase
**S1**	(MH "Child+") OR (MH "Adolescence+") OR (MH "Young Adult") OR (MH "Adolescent Psychology")	Expanders - Apply equivalent subjects<br>Search modes - Boolean/Phrase

Global Health search

#	Query	Limiters/Expanders
**S17**	S4 AND S8 AND S12 AND S16	Limiters - Publication Year: 20000101-<br>Expanders - Apply equivalent subjects<br>Search modes - Boolean/Phrase
**S16**	S13 OR S14 OR S15	Expanders - Apply equivalent subjects

		Search modes - Boolean/Phrase
**S15**	AB "sub-saharan Africa" OR AB Angola OR AB Benin OR AB Botswana OR AB "Burkina Faso" OR AB Burundi OR AB Cameroon OR AB "Cape Verde" OR AB "Central African Republic" OR AB Chad OR AB Comoros OR AB "Republic of the Congo" OR AB "Democratic Republic of the Congo" OR AB "Cote d'Ivoire" OR AB "Ivory Coast" OR AB Djibouti OR AB "Equatorial Guinea" OR AB Eritrea OR AB Eswatini OR AB Ethiopia OR AB Gabon OR AB "The Gambia" OR AB Ghana OR AB Guinea OR AB "Guinea-Bissau" OR AB Kenya OR AB Lesotho OR AB Liberia OR AB Madagascar OR AB Malawi OR AB Mali OR AB Mauritania OR AB Mauritius OR AB Mozambique OR AB Namibia OR AB Niger OR AB Nigeria OR AB Rwanda OR AB "Sao Tome and Principe" OR AB Senegal OR AB Seychelles OR AB "Sierra Leone" OR AB "South Africa" OR AB "South Sudan" OR AB Sudan OR AB Swaziland OR AB Tanzania OR AB Togo OR AB Uganda OR AB Zambia OR AB Zimbabwe	Expanders - Apply equivalent subjects<br>Search modes - Boolean/Phrase
**S14**	TI "sub-saharan Africa" OR TI Angola OR TI Benin OR TI Botswana OR TI "Burkina Faso" OR TI Burundi OR TI Cameroon OR TI "Cape Verde" OR TI "Central African Republic" OR TI Chad OR TI Comoros OR TI "Republic of the Congo" OR TI "Democratic Republic of the Congo" OR TI "Cote d'Ivoire" OR TI "Ivory Coast" OR TI Djibouti OR TI "Equatorial Guinea" OR TI Eritrea OR TI Eswatini OR TI Ethiopia OR TI Gabon OR TI "The Gambia" OR TI Ghana OR TI Guinea OR TI "Guinea-Bissau" OR TI Kenya OR TI Lesotho OR TI Liberia OR TI Madagascar OR TI Malawi OR TI Mali OR TI Mauritania OR TI Mauritius OR TI Mozambique OR TI Namibia OR TI Niger OR TI Nigeria OR TI Rwanda OR TI "Sao Tome and Principe" OR TI Senegal OR TI Seychelles OR TI "Sierra Leone" OR TI "South Africa" OR TI "South Sudan" OR TI Sudan OR TI Swaziland OR TI Tanzania OR TI Togo OR TI Uganda OR TI Zambia OR TI Zimbabwe	Expanders - Apply equivalent subjects<br>Search modes - Boolean/Phrase
**S13**	DE "Africa South of Sahara" OR DE "Angola" OR DE "Benin" AND DE "Botswana" OR DE "Burkina Faso" AND DE "Burundi" OR DE "Cameroon" OR DE "Cape Verde" OR DE "Central African Republic" OR DE "Chad" OR DE "Comoros" OR DE "Grande Comore" OR DE "Moheli" OR DE "Anjouan" OR DE "Congo" OR DE "Congo Democratic Republic" OR DE "Cote d'Ivoire" OR DE "Djibouti" OR DE "Guinea" OR DE "Equatorial Guinea" OR DE "Guinea-Bissau" OR DE "Kenya" OR DE "Lesotho" OR DE "Liberia" OR DE "Madagascar" OR DE "Malawi" OR DE "Mali" OR DE "Mauritania" OR DE "Mauritius" OR DE "Mozambique" OR DE "Niger" OR DE "Nigeria" OR DE "Rwanda" OR DE "Sao Tome and Principe" OR DE "Senegal" OR DE "Seychelles" OR DE "Aldabra" OR DE "Sierra Leone" OR DE "Namibia" OR DE "South Africa" OR DE "Sudan" OR DE "Southern Sudan" OR DE "Swaziland" OR DE "Tanzania" OR DE "Zanzibar" OR DE "Togo" OR DE "Uganda" OR DE "Zambia" OR DE "Zimbabwe"	Expanders - Apply equivalent subjects<br>Search modes - Boolean/Phrase

**S12**	S9 OR S10 OR S11	Expanders - Apply equivalent subjects<br>Search modes - Boolean/Phrase
**S11**	AB promot* OR AB prevent* OR AB awareness OR AB campaign OR AB training OR AB education OR AB intervention	Expanders - Apply equivalent subjects<br>Search modes - Boolean/Phrase
**S10**	TI promot* OR TI prevent* OR TI awareness OR TI campaign OR TI training OR TI education OR TI intervention	Expanders - Apply equivalent subjects<br>Search modes - Boolean/Phrase
**S9**	(DE "health promotion") OR (DE "disease prevention" OR DE "prophylaxis")	Expanders - Apply equivalent subjects<br>Search modes - Boolean/Phrase
**S8**	S5 OR S6 OR S7	Expanders - Apply equivalent subjects<br>Search modes - Boolean/Phrase
**S7**	AB "mental health" OR AB "mental health literacy" OR AB psychological OR AB psychosocial OR AB wellbeing OR AB emotional OR AB coping OR AB resilien* OR AB "mental health stigma" OR AB distress	Expanders - Apply equivalent subjects<br>Search modes - Boolean/Phrase
**S6**	TI "mental health" OR TI "mental health literacy" OR TI psychological OR TI psychosocial OR TI wellbeing OR TI emotional OR TI coping OR TI resilien* OR TI "mental health stigma" OR TI distress	Expanders - Apply equivalent subjects<br>Search modes - Boolean/Phrase
**S5**	(DE "mental health") OR (DE "mental stress")	Expanders - Apply equivalent subjects<br>Search modes - Boolean/Phrase
**S4**	S1 OR S2 OR S3	Expanders - Apply equivalent subjects<br>Search modes - Boolean/Phrase
**S3**	AB child* OR AB adolescen* OR AB teen* OR AB juvenile OR AB youth OR AB "young adult" OR AB “young person” OR AB “young people”	Expanders - Apply equivalent subjects<br>Search modes - Boolean/Phrase
**S2**	TI child* OR TI adolescen* OR TI teen* OR TI juvenile OR TI youth OR TI "young adult" OR TI “young person” OR TI “young people”	Expanders - Apply equivalent subjects<br>Search modes - Boolean/Phrase
**S1**	(DE "children") OR (DE "adolescents") OR (DE "young adults")	Expanders - Apply equivalent subjects<br>Search modes - Boolean/Phrase

**#**	**Query**	**Limiters/Expanders**

**S17**	S4 AND S8 AND S12 AND S16	Limiters - Publication Year: 20000101-<br>Expanders - Apply equivalent subjects<br>Search modes - Boolean/Phrase
**S16**	S13 OR S14 OR S15	Expanders - Apply equivalent subjects<br>Search modes - Boolean/Phrase
**S15**	AB "sub-saharan Africa” OR AB Angola OR AB Benin OR AB Botswana OR AB “Burkina Faso” OR AB Burundi OR AB Cameroon OR AB “Cape Verde” OR AB “Central African Republic” OR AB Chad OR AB Comoros OR AB “Republic of the Congo” OR AB “Democratic Republic of the Congo” OR AB "Cote d'Ivoire" OR AB "Ivory Coast" OR AB Djibouti OR AB "Equatorial Guinea" OR AB Eritrea OR AB Eswatini OR AB Ethiopia OR AB Gabon OR AB “The Gambia” OR AB Ghana OR AB Guinea OR AB “Guinea-Bissau” OR AB Kenya OR AB Lesotho OR AB Liberia OR AB Madagascar OR AB Malawi OR AB Mali OR AB Mauritania OR AB Mauritius OR AB Mozambique OR AB Namibia OR AB Niger OR AB Nigeria OR AB Rwanda OR AB “Sao Tome and Principe” OR AB Senegal OR AB Seychelles OR AB "Sierra Leone" OR AB “South Africa” OR AB “South Sudan” OR AB Sudan OR AB Swaziland OR AB Tanzania OR AB Togo OR AB Uganda OR AB Zambia OR AB Zimbabwe	Expanders - Apply equivalent subjects<br>Search modes - Boolean/Phrase
**S14**	TI "sub-saharan Africa” OR TI Angola OR TI Benin OR TI Botswana OR TI “Burkina Faso” OR TI Burundi OR TI Cameroon OR TI “Cape Verde” OR TI “Central African Republic” OR TI Chad OR TI Comoros OR TI “Republic of the Congo” OR TI “Democratic Republic of the Congo” OR TI "Cote d'Ivoire" OR TI "Ivory Coast" OR TI Djibouti OR TI "Equatorial Guinea" OR TI Eritrea OR TI Eswatini OR TI Ethiopia OR TI Gabon OR TI “The Gambia” OR TI Ghana OR TI Guinea OR TI “Guinea-Bissau” OR TI Kenya OR TI Lesotho OR TI Liberia OR TI Madagascar OR TI Malawi OR TI Mali OR TI Mauritania OR TI Mauritius OR TI Mozambique OR TI Namibia OR TI Niger OR TI Nigeria OR TI Rwanda OR TI “Sao Tome and Principe” OR TI Senegal OR TI Seychelles OR TI "Sierra Leone" OR TI “South Africa” OR TI “South Sudan” OR TI Sudan OR TI Swaziland OR TI Tanzania OR TI Togo OR TI Uganda OR TI Zambia OR TI Zimbabwe	Expanders - Apply equivalent subjects<br>Search modes - Boolean/Phrase
**S13**	DE "Africa South of Sahara" OR DE "Angola" OR DE "Benin" AND DE "Botswana" OR DE "Burkina Faso" AND DE "Burundi" OR DE "Cameroon" OR DE "Cape Verde" OR DE "Central African Republic" OR DE "Chad" OR DE "Comoros" OR DE "Grande Comore" OR DE "Moheli" OR DE "Anjouan" OR DE "Congo" OR DE "Congo Democratic Republic" OR DE "Cote d'Ivoire" OR DE "Djibouti" OR DE "Guinea" OR DE "Equatorial Guinea" OR DE "Guinea-Bissau" OR DE "Kenya" OR DE "Lesotho" OR DE "Liberia" OR DE "Madagascar" OR	Expanders - Apply equivalent subjects<br>Search modes - Boolean/Phrase

	DE "Malawi" OR DE "Mali" OR DE "Mauritania" OR DE "Mauritius" OR DE "Mozambique" OR DE "Niger" OR DE "Nigeria" OR DE "Rwanda" OR DE "Sao Tome and Principe" OR DE "Senegal" OR DE "Seychelles" OR DE "Aldabra" OR DE "Sierra Leone" OR DE "Namibia" OR DE "South Africa" OR DE "Sudan" OR DE "Southern Sudan" OR DE "Swaziland" OR DE "Tanzania" OR DE "Zanzibar" OR DE "Togo" OR DE "Uganda" OR DE "Zambia" OR DE "Zimbabwe"	
**S12**	S9 OR S10 OR S11	Expanders - Apply equivalent subjects<br>Search modes - Boolean/Phrase
**S11**	AB promot* OR AB prevent* OR AB awareness OR AB campaign OR AB training OR AB education OR AB intervention	Expanders - Apply equivalent subjects<br>Search modes - Boolean/Phrase
**S10**	TI promot* OR TI prevent* OR TI awareness OR TI campaign OR TI training OR TI education OR TI intervention	Expanders - Apply equivalent subjects<br>Search modes - Boolean/Phrase
**S9**	(DE "health promotion") OR (DE "disease prevention" OR DE "prophylaxis")	Expanders - Apply equivalent subjects<br>Search modes - Boolean/Phrase
**S8**	S5 OR S6 OR S7	Expanders - Apply equivalent subjects<br>Search modes - Boolean/Phrase
**S7**	AB "mental health" OR AB "mental health literacy" OR AB psychological OR AB psychosocial OR AB wellbeing OR AB emotional OR AB coping OR AB resilien* OR AB "mental health stigma" OR AB distress	Expanders - Apply equivalent subjects<br>Search modes - Boolean/Phrase
**S6**	TI "mental health" OR TI "mental health literacy" OR TI psychological OR TI psychosocial OR TI wellbeing OR TI emotional OR TI coping OR TI resilien* OR TI "mental health stigma" OR TI distress	Expanders - Apply equivalent subjects<br>Search modes - Boolean/Phrase
**S5**	(DE "mental health") OR (DE "mental stress")	Expanders - Apply equivalent subjects<br>Search modes - Boolean/Phrase
**S4**	S1 OR S2 OR S3	Expanders - Apply equivalent subjects<br>Search modes - Boolean/Phrase
**S3**	AB child* OR AB adolescen* OR AB teen* OR AB juvenile OR AB youth OR AB "young adult" OR AB “young person” OR AB “young people”	Expanders - Apply equivalent subjects<br>Search modes - Boolean/Phrase
**S2**	TI child* OR TI adolescen* OR TI teen* OR TI juvenile OR TI youth OR TI "young adult" OR TI “young person” OR TI “young people”	Expanders - Apply equivalent subjects<br>Search modes - Boolean/Phrase

**S1**	(DE "children") OR (DE "adolescents") OR (DE "young adults")	Expanders - Apply equivalent subjects<br>Search modes - Boolean/Phrase

PsychInfo Search

#	Query	Limiters/Expanders
**S21**	S5 AND S10 AND S15 AND S18	Limiters - Date of Publication: 20000101-<br>Expanders - Apply equivalent subjects<br>Search modes - Boolean/Phrase
**S18**	S16 OR S17	Expanders - Apply equivalent subjects<br>Search modes - Boolean/Phrase
**S17**	AB "sub-saharan Africa” OR AB Angola OR AB Benin OR AB Botswana OR AB “Burkina Faso” OR AB Burundi OR AB Cameroon OR AB “Cape Verde” OR AB “Central African Republic” OR AB Chad OR AB Comoros OR AB “Republic of the Congo” OR AB “Democratic Republic of the Congo” OR AB "Cote d'Ivoire" OR AB "Ivory Coast" OR AB Djibouti OR AB "Equatorial Guinea" OR AB Eritrea OR AB Eswatini OR AB Ethiopia OR AB Gabon OR AB “The Gambia” OR AB Ghana OR AB Guinea OR AB “Guinea-Bissau” OR AB Kenya OR AB Lesotho OR AB Liberia OR AB Madagascar OR AB Malawi OR AB Mali OR AB Mauritania OR AB Mauritius OR AB Mozambique OR AB Namibia OR AB Niger OR AB Nigeria OR AB Rwanda OR AB “Sao Tome and Principe” OR AB Senegal OR AB Seychelles OR AB "Sierra Leone" OR AB “South Africa” OR AB “South Sudan” OR AB Sudan OR AB Swaziland OR AB Tanzania OR AB Togo OR AB Uganda OR AB Zambia OR AB Zimbabwe	Expanders - Apply equivalent subjects<br>Search modes - Boolean/Phrase
**S16**	TI "sub-saharan Africa” OR TI Angola OR TI Benin OR TI Botswana OR TI “Burkina Faso” OR TI Burundi OR TI Cameroon OR TI “Cape Verde” OR TI “Central African Republic” OR TI Chad OR TI Comoros OR TI “Republic of the Congo” OR TI “Democratic Republic of the Congo” OR TI "Cote d'Ivoire" OR TI "Ivory Coast" OR TI Djibouti OR TI "Equatorial Guinea" OR TI Eritrea OR TI Eswatini OR TI Ethiopia OR TI Gabon OR TI “The Gambia” OR TI Ghana OR TI Guinea OR TI “Guinea-Bissau” OR TI Kenya OR TI Lesotho OR TI Liberia OR TI Madagascar OR TI Malawi OR TI Mali OR TI Mauritania OR TI Mauritius OR TI Mozambique OR TI Namibia OR TI Niger OR TI Nigeria OR TI Rwanda OR TI “Sao Tome and Principe” OR TI Senegal OR TI Seychelles OR TI "Sierra Leone" OR TI “South Africa” OR TI “South Sudan” OR TI Sudan OR TI Swaziland OR TI Tanzania OR TI Togo OR TI Uganda OR TI Zambia OR TI Zimbabwe	Expanders - Apply equivalent subjects<br>Search modes - Boolean/Phrase

**S15**	S11 OR S14	Expanders - Apply equivalent subjects<br>Search modes - Boolean/Phrase
**S14**	S12 OR S13	Expanders - Apply equivalent subjects<br>Search modes - Boolean/Phrase
**S13**	AB child* OR AB adolescen* OR AB teen* OR AB juvenile OR AB youth OR AB "young adult" OR AB “young person” OR AB “young people”	Expanders - Apply equivalent subjects<br>Search modes - Boolean/Phrase
**S12**	TI child* OR TI adolescen* OR TI teen* OR TI juvenile OR TI youth OR TI "young adult" OR TI “young person” OR TI “young people”	Expanders - Apply equivalent subjects<br>Search modes - Boolean/Phrase
**S11**	Youth mental health.sh OR adolescent development.sh OR childhood development.sh OR adolescent psychology.sh OR child psychology.sh	Expanders - Apply equivalent subjects<br>Search modes - Boolean/Phrase
**S10**	S6 OR S9	Expanders - Apply equivalent subjects<br>Search modes - Boolean/Phrase
**S9**	S7 OR S8	Expanders - Apply equivalent subjects<br>Search modes - Boolean/Phrase
**S8**	AB promot* OR AB prevent* OR AB awareness OR AB campaign OR AB training OR AB education OR AB intervention	Expanders - Apply equivalent subjects<br>Search modes - Boolean/Phrase
**S7**	TI promot* OR TI prevent* OR TI awareness OR TI campaign OR TI training OR TI education OR TI intervention	Expanders - Apply equivalent subjects<br>Search modes - Boolean/Phrase
**S6**	Preventive mental health services.sh OR mental health programs.sh OR school based mental health services.sh OR OR community psychiatry.sh OR community mental health services.sh OR mental health education.sh OR strengths-based interventions.sh OR community psychology.sh OR program development.sh OR intervention.sh OR psychoeducation.sh OR self-help techniques.sh OR early intervention.sh OR prevention.sh OR stress management.sh OR public health campaigns.sh	Expanders - Apply equivalent subjects<br>Search modes - Boolean/Phrase
**S5**	S1 OR S4	Expanders - Apply equivalent subjects<br>Search modes - Boolean/Phrase
**S4**	S2 OR S3	Expanders - Apply equivalent subjects

		Search modes - Boolean/Phrase
**S3**	AB "mental health" OR AB "mental health literacy" OR AB psychological OR AB psychosocial OR AB wellbeing OR AB emotional OR AB coping OR AB resilien* OR AB "mental health stigma" OR AB distress OR anxiety OR depression	Expanders - Apply equivalent subjects<br>Search modes - Boolean/Phrase
**S2**	TI "mental health" OR TI "mental health literacy" OR TI psychological OR TI psychosocial OR TI wellbeing OR TI emotional OR TI coping OR TI resilien* OR TI "mental health stigma" OR TI distress	Expanders - Apply equivalent subjects<br>Search modes - Boolean/Phrase
**S1**	emotional health.sh OR global mental health.sh OR public mental health.sh OR psychological stress.sh OR distress.sh OR mental health stigma.sh OR resilience(psychological).sh OR coping behavior.sh OR mental health literacy.sh OR mental health.sh OR wellbeing.sh OR mental disorders.sh	Expanders - Apply equivalent subjects<br>Search modes - Boolean/Phrase

COCHRANE DATABASE SEARCH

Cochrane was searched with the terms “mental health” AND “Africa”, “mental health prevention” AND “Africa”, and “psychological” AND “Africa”.
